# A Small Interfering RNA Cocktail Targeting the Nucleoprotein and Large Protein Genes Suppresses Borna Disease Virus Infection

**DOI:** 10.3389/fmicb.2019.02781

**Published:** 2019-11-29

**Authors:** Da Teng, Shunsuke Obika, Keiji Ueda, Tomoyuki Honda

**Affiliations:** Division of Virology, Department of Microbiology and Immunology, Osaka University Graduate School of Medicine, Osaka, Japan

**Keywords:** antiviral, Borna disease virus, neuronal cells, replication, siRNA, therapy

## Abstract

Recently, Borna disease virus (BoDV-1)-related fatal encephalitis human cases have been reported, which highlights the potential of BoDV-1 to cause fatal human diseases. To protect the infected brain from lethal damage, it is critical to control BoDV-1 as quickly as possible. At present, antivirals against BoDV-1 are limited, and therefore, novel types of antivirals are needed. Here, we developed a novel treatment using small interfering RNAs (siRNAs) against BoDV-1. We screened several siRNAs targeting the viral N, M, and L genes for BoDV-1-reducing activity. Among the screened candidates, we chose two siRNAs that efficiently decreased the BoDV-1 load in persistently BoDV-1-infected cells to prepare a siRNA cocktail (TD-Borna) for BoDV-1 treatment. TD-Borna successfully reduced the BoDV-1 load without enhancing the risk of emergence of escape mutants. The combination of TD-Borna and T-705, a previously reported antiviral agent against bornaviruses, decreased the BoDV-1 load more efficiently than TD-Borna or T-705 alone. Furthermore, TD-Borna efficiently decreased the BoDV-1 load in BoDV-1-infected neuron-derived cells, in which T-705 did not decrease the viral load. Overall, we developed a novel antiviral candidate against BoDV-1, TD-Borna, that can be used in combination with T-705 and is effective against BoDV-1 in neuron-derived cells, in which T-705 is less effective. Considering that BoDV-1 is highly neurotropic, TD-Borna can offer a promising option to improve the outcome of BoDV-1 infection.

## Introduction

Recently, fatal human encephalitis cases caused by bornavirus infections have been sporadically reported in Europe (Hoffmann et al., 2015; Korn et al., 2018; Schlottau et al., 2018). Additionally, although it is still controversial, several studies have reported potential human cases of bornavirus infection in various psychiatric disorders (Nakamura et al., 2000; Honda et al., 2017b, 2018). Indeed, there are a few human case reports showing remission of symptoms after treatment with antivirals against bornaviruses, although the causative relationship is unclear (Matsunaga et al., 2018). So far, ribavirin and T-705 (favipiravir) are nominated as antivirals against bornaviruses (Mizutani et al., 1998; Musser et al., 2015; Reuter et al., 2016; Tokunaga et al., 2017). However, to increase the chance of an improved outcome of a potential bornavirus infection, the development of novel therapeutic strategies that can be used in combination with ribavirin or T-705 is needed.

Borna disease virus (BoDV-1) is a member of *Mammalian 1 bornavirus* of the genus *Bornavirus* (Amarasinghe et al., 2019) and a bornavirus prototype that infects various animal species (Staeheli et al., 2000). As the name indicates, it is a causative agent of Borna disease, a fatal encephalomyelitis in horses and sheep (Staeheli et al., 2000). BoDV-1 is a non-segmented, negative-strand RNA virus that encodes at least six viral proteins, nucleoprotein (N), phosphoprotein (P), X, matrix (M), glycoprotein (G), and large protein (L) (Tomonaga et al., 2002; Honda and Tomonaga, 2013). BoDV-1 genomic RNA (gRNA) is encapsidated by the N protein. The encapsidated BoDV-1 gRNA and the viral RNA-dependent RNA polymerase complex, which consists of the P and L proteins, form the BoDV-1 ribonucleoprotein complex (RNP) (Tomonaga et al., 2002; Honda and Tomonaga, 2013). BoDV-1 RNP is the replication unit of the virus. During replication, the BoDV-1 polymerase complex synthesizes BoDV-1 antigenomic RNA, which in turn acts as a template for BoDV-1 gRNA synthesis. The M protein plays a role in viral particle assembly and budding (Kraus et al., 2001). The G protein is responsible for viral entry (Bajramovic et al., 2003; Honda et al., 2009).

RNA interference (RNAi) is a practical strategy to treat various types of viruses, such as Ebola virus (Geisbert et al., 2010), influenza virus (McMillen et al., 2016), dengue virus (Paul et al., 2014), and rabies virus (Meshram et al., 2013), because of the ease of the design and manufacturing of small interfering RNAs (siRNAs). Furthermore, fundamental knowledge of siRNA molecules, such as the pharmacological dynamics of siRNAs *in vivo*, can be applied to any siRNA-derived drug regardless of the sequence of the designed siRNAs. This potentially facilitates the processes from siRNA design to the practical use of siRNA-derived drugs. Thus, siRNAs targeting viral RNAs can be promising alternatives to current antivirals.

Here, we designed siRNAs targeting BoDV-1 mRNAs and evaluated whether the siRNAs could suppress BoDV-1 infection. We chose BoDV-1 N and M/G/L mRNAs to be targeted by the siRNAs because both encode proteins, i.e., the N and L proteins, indispensable for BoDV replication. We successfully obtained siRNAs that attenuated BoDV-1 infection. Using these siRNAs, we developed a novel BoDV-1 therapeutic strategy that could improve the outcome of BoDV-1-related diseases.

## Materials and Methods

### Cells

293T cells (a human embryonic kidney cell line) and Vero cells (a monkey kidney cell line) were cultured in Dulbecco’s modified Eagle’s medium (DMEM) supplemented with 5% fetal bovine serum (FBS). SH-SY5Y cells (a human neuroblastoma cell line) were cultured in DMEM:Ham’s F12 supplemented with 10% FBS and 1% non-essential amino acids (NEAA). N2a cells (a mouse neuroblastoma cell line) were cultured in DMEM supplemented with 10% FBS. 293T, SH-SY5Y, and N2a cells were infected with huP2Br (Nakamura et al., 2000), huP2Br, and HOT6 (Honda et al., 2017a) strains of BoDV-1, respectively. These cells were passaged for >2 months to obtain persistently BoDV-1-infected 293T (293T/BoDV), SH-SY5Y (SH-SY5Y/BoDV), and N2a (N2a/BoDV) cells.

### siRNAs

The sequences of the siRNAs used in this study were as follows:

si-Control, MISSION Negative control (Sigma–Aldrich, St. Louis, MO, United States)
si-BoDV N #1, 5′-GUU AAU CCA AUC UAU AGC CUC-3′ and 5′-GGC UAU AGA UUG GAU UAA CGG-3′
si-BoDV N #2, 5′-UCU UAC UCC AGU AAA ACG CUG-3′ and 5′-GCG UUU UAC UGG AGU AAG AAG-3′
si-BoDV M #3, 5′-AAA GAA AUG GGA UGU UAA GGA-3′ and 5′-CUU AAC AUC CCA UUU CUU UCA-3′
si-BoDV L #4, 5′-UCA AGU UGA AGC AGU UUU UUU-3′ and 5′-AAA AAC UGC UUC AAC UUG ACC-3′.

### Transfection

The cells were transiently transfected using Lipofectamine RNAiMAX (Invitrogen, Carlsbad, CA, United States) at a final concentration of 10 nM for the siRNAs. siRNAs were repeatedly transfected into the cell once a week for long-term treatment.

### T-705 and Ribavirin

T-705 (favipiravir) and ribavirin were purchased from Toronto Research Chemicals (North York, ON, Canada) and Selleck Chemicals (Houston, TX, United States), respectively. The cells were treated with T-705 at a final concentration of 400 μM, at which no cytotoxicity but substantial anti-bornavirus activity was detected previously (Tokunaga et al., 2017). Ribavirin was used at a final concentration of 10 μM.

### Real-Time RT-PCR

Real-time RT-PCR was conducted, as described previously, with some modifications (Hayashi et al., 2009; Nishikawa et al., 2018; Nakayama et al., 2019). Briefly, total RNA was extracted from the indicated cells and reverse-transcribed using a Verso cDNA Synthesis Kit (Thermo Fisher Scientific, Waltham, United States) according to the manufacturer’s instruction. Quantitative RT-PCR (RT-qPCR) assays for human GAPDH, mouse GAPDH, and BoDV-1 N mRNAs were carried out using Fast SYBR Green Master Mix (Thermo Fisher Scientific, Waltham, United States) and gene-specific primers with QuantStudio 6 (Thermo Fisher Scientific, Waltham, United States) or CFX Connect Real-Time PCR Detection System (Bio-Rad Laboratories, Inc., Hercules, CA, United States). RT-qPCR assays for BoDV-1 L mRNA and BoDV-1 gRNA were carried out using Luna Universal Probe qPCR Master Mix (New England Biolabs, Ipswich, United States) and the BoDV-1-specific primers and probe. The gene-specific primers and probes used in this study were shown in Supplementary Table S1.

### Western Blotting

The cells were lysed in sodium dodecyl sulfate (SDS) sample buffer. Total cell lysate was subjected to SDS-PAGE and transferred onto polyvinylidene difluoride membranes (Millipore, Bedford, MA, United States). The membrane was blotted with mouse anti-N (HN132) and mouse anti-tubulin (Wako Pure Chemical Industries, Osaka, Japan) antibodies. Band intensities for the N protein were measured using Image J software and normalized with those of corresponding bands of tubulin.

### Virus Preparation and *de novo* Infection

Infectious viruses were prepared as described previously (Daito et al., 2011; Yamamoto et al., 2019). Briefly, BoDV-1-infected cells were sonicated in DMEM supplemented with 2% FBS. After centrifugation, the supernatants were collected as virus stocks. Then, Vero cells were infected with the viruses and incubated at 37°C for 3 days. The virus titers (focus forming units/ml) were determined by immunofluorescence analysis (Nakayama et al., 2019; Yamamoto et al., 2019) using anti-N (HB03) and anti-P (HP062) antibodies.

### Sequencing of the Target Sites

Amplicons of the N or L gene were generated using the N- or L-target-specific primers and then cloned into a pCMV-Myc plasmid (Takara Bio, Shiga, Japan). Twenty clones for each amplicon were sequenced. The primers used in this study were shown in Supplementary Table S1.

### Statistics

Statistical significance was assessed using a two-tailed Student’s *t*-test and analysis of variance (ANOVA).

## Results

### Reduction in the BoDV-1 Load by siRNAs Targeting N and M/G/L mRNAs

**FIGURE 1:**
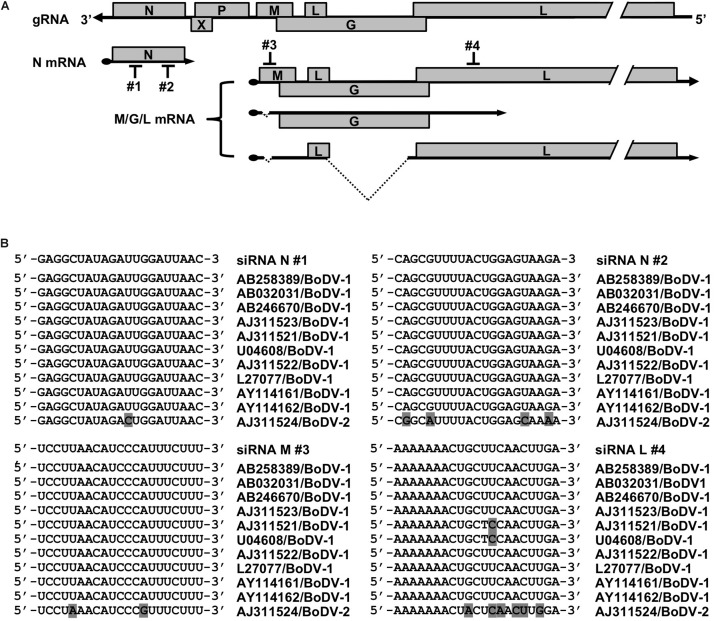
siRNAs designed to target BoDV-1 genes. **(A)** Schematic view of the BoDV-1 genome and mRNAs with the designed siRNAs. **(B)** Alignment of the siRNA target sequences and the mRNA sequences of various BoDV-1 and BoDV-2 strains.

**FIGURE 2:**
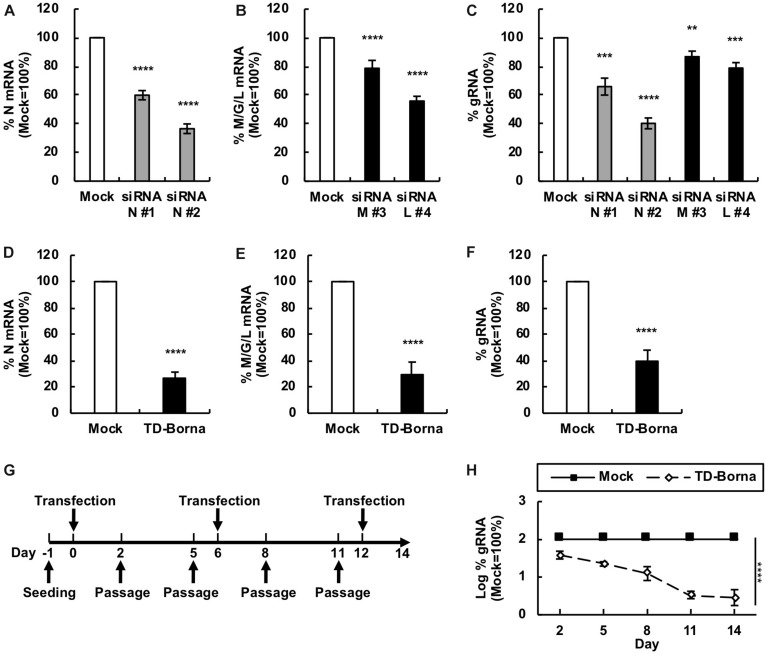
Reduction in the BoDV-1 load by siRNAs targeting N and M/G/L mRNAs. **(A,B)** Effects of siRNAs targeting BoDV-1 mRNAs on BoDV-1 mRNA amounts. 293T/BoDV cells were treated with the indicated siRNA for 2 days. The amounts of N **(A)** and M/G/L **(B)** mRNAs in 293T/BoDV cells were determined by RT-qPCR analyses. **(C)** Effects of siRNAs targeting BoDV-1 mRNAs on BoDV-1 replication. The amount of BoDV-1 gRNA in 293T/BoDV cells was determined by RT-qPCR analyses. **(D,E)** Effects of the siRNA cocktail (TD-Borna) targeting BoDV-1 N and M/G/L mRNAs on BoDV-1 mRNAs. 293T/BoDV cells were treated with TD-Borna for 2 days. The amounts of N **(D)** and M/G/L **(E)** mRNAs in 293T/BoDV cells were determined by RT-qPCR analyses. **(F)** Effects of TD-Borna on BoDV-1 replication. The amount of BoDV-1 gRNA in 293T/BoDV cells was determined by RT-qPCR analyses. **(G)** Protocol for long-term TD-Borna treatment in 293T/BoDV cells. 293T/BoDV cells were seeded in 12-well plates (2 × 10^5^ cells/well) on Day –1. TD-Borna was transfected on Days 0, 6, and 12. The cells were passaged into new 12-well plates (2 × 10^5^ cells/well) every 3 days. **(H)** Effects of long-term TD-Borna treatment on BoDV-1 replication. The amount of BoDV-1 gRNA in 293T/BoDV cells was determined by RT-qPCR analyses. Mock, the scrambled siRNA-treated control. Values are expressed as the mean ± SE of three independent experiments. ^∗∗^*P* < 0.01, ^∗∗∗^*P* < 0.005, and ^****^P < 0.001 (vs. mock).

To develop a novel therapeutic tool for the treatment of BoDV-1 infection, we designed siRNAs targeting BoDV-1 mRNAs (Figure 1). BoDV-1 has three major transcripts encoding viral proteins, i.e., N mRNA, X/P mRNA, and M/G/L mRNA. Among them, N is essential for the encapsidation of BoDV gRNA, and L is an essential RNA-dependent RNA polymerase required for viral RNA synthesis. Therefore, we chose two targeting regions each, conserved among various BoDV-1 strains, for N and M/G/L mRNAs (#1 and #2 for N mRNA; #3 and #4 for M/G/L mRNA) (Figure 1). When the siRNAs were transfected into 293T cells persistently infected with BoDV-1 (293T/BoDV), the siRNAs successfully downregulated their respective target mRNAs (Figures 2A,B). Because BoDV-1 requires the N and L proteins to amplify gRNA, downregulation of these mRNAs is also expected to downregulate BoDV-1 gRNA. We therefore evaluated the effect of these siRNAs on the amount of BoDV-1 gRNA. As expected, the siRNAs targeting N or M/G/L mRNA successfully decreased BoDV-1 gRNA (Figure 2C).

### Reduction of the BoDV-1 Load by a siRNA Cocktail, TD-Borna

To reduce the risk of the emergence of escape mutants during siRNA treatment, we sought to use the siRNAs in combination. We prepared two siRNA cocktails: TD-Borna and TD-Borna-4Mix, a cocktail of siRNAs #2 and #4 and a cocktail of all four siRNAs, respectively. TD-Borna decreased both N and M/G/L mRNAs by ∼70% (Figures 2D,E), while TD-Borna-4Mix decreased the mRNAs by ∼50% (Supplementary Figures S1A,B). We confirmed that both cocktails also decreased viral gene expression at the protein level (Supplementary Figure S2). TD-Borna downregulated the amount of the N protein more efficiently than TD-Borna-4Mix (Supplementary Figure S2B), consistent with the amounts of BoDV-1 RNAs. Furthermore, TD-Borna decreased the BoDV-1 load by ∼60% (Figure 2F), whereas TD-Borna-4Mix did by ∼40% (Supplementary Figure S1C). These results indicate that TD-Borna downregulates the BoDV-1 load more efficiently than TD-Borna-4Mix. We further confirmed the inhibitory effect of TD-Borna on the production of infectious BoDV-1 particles (Supplementary Figure S3). Based on these observations, we chose to use TD-Borna for further study.

We next evaluated whether TD-Borna could control BoDV-1 infection for a long period of time. To this end, we transfected TD-Borna into 293T/BoDV cells once a week and measured the BoDV-1 load for 2 weeks (Figure 2G). As expected, TD-Borna efficiently decreased the BoDV-1 load over time to the background level (Figure 2H). We then evaluated the emergence of escape mutants by sequencing the target sequences of TD-Borna in BoDV-1 gRNA at 2 weeks after treatment with scrambled siRNA, siRNA N #2, siRNA L #4, or TD-Borna. The average mutation frequencies of the N target sites in cells treated with scrambled siRNA, siRNA N #2, and TD-Borna were 0.12, 0.72, and 0.14% per nucleotide position, respectively. Those of the L target sites in cells treated with scrambled siRNA, siRNA L #4, and TD-Borna were 0.24, 0.69, and 0.34% per nucleotide position, respectively. These results suggest that TD-Borna treatment might be less prone to produce mutations than single siRNA treatment. Taken together, TD-Borna can be a novel safer option for the long-term treatment of BoDV-1 infection.

### Reduction in the BoDV-1 Load by the Combined Use of T-705 and TD-Borna

**FIGURE 3:**
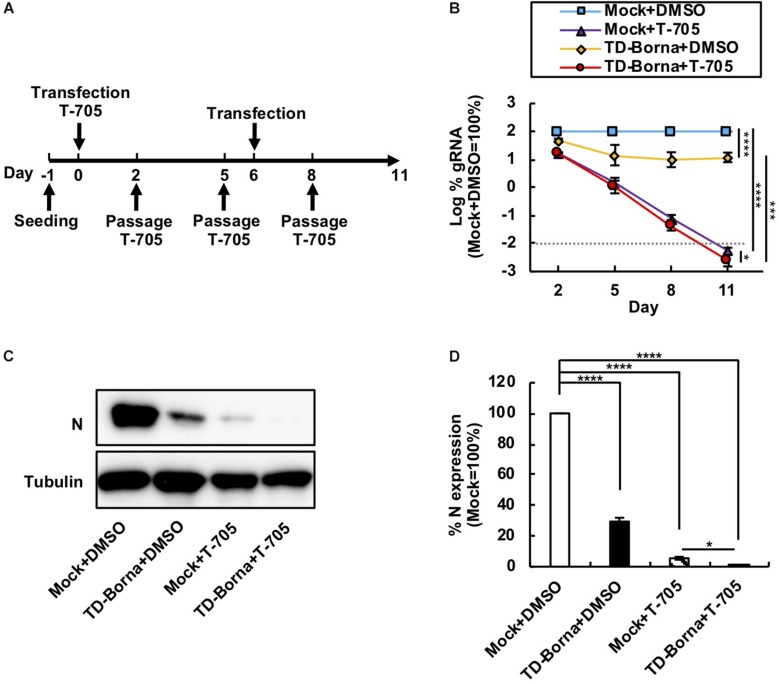
Reduction in the BoDV-1 load by the combined use of T-705 and TD-Borna. **(A)** Protocol for long-term treatment using T-705 and TD-Borna in 293T/BoDV cells. 293T/BoDV cells were seeded in 12-well plates (2 × 10^5^ cells/well) on Day –1. TD-Borna was transfected on Days 0 and 6. On Day 0, T-705 was added at 1 h after the transfection. The cells were passaged into new 12-well plates (2 × 10^5^ cells/well) with or without T-705 on Days 2, 5, and 8. **(B)** Effects of long-term treatment with T-705 and TD-Borna on BoDV-1 replication. The amount of BoDV-1 gRNA in 293T/BoDV cells was determined by RT-qPCR analyses. **(C)** The amount of the N protein in 293T/BoDV cells treated with T-705 and TD-Borna. 293T/BoDV cells were treated as indicated in panel **(A)**. After 5 days of treatment, the amount of the N protein was determined by western blotting using anti-N and anti-tubulin antibodies. **(D)** Quantification of the amount of the N protein in panel **(C)**. The band intensity of the N protein in each sample was normalized with that of tubulin. Mock, the scrambled siRNA-treated control. Values are expressed as the mean ± SE of three independent experiments. ^∗^*P* < 0.05, ^∗∗∗^*P* < 0.005, and ^****^P < 0.001.

Considering that BoDV infects neurons and that neuronal damage is hard to recover from, it is important to decrease the BoDV load as quickly as possible. We have previously demonstrated that T-705 efficiently inhibits BoDV replication without any cytotoxicity (Tokunaga et al., 2017). Because T-705 and TD-Borna have different mechanisms to downregulate the BoDV-1 load, we reasoned that a combined use of T-705 and TD-Borna would have a more prominent effect against BoDV-1. To evaluate the effect of the combined use, we transfected TD-Borna into 293T/BoDV cells in the presence or absence of T-705 (Figure 3A). Both T-705 and TD-Borna efficiently downregulated the BoDV-1 load (Figure 3B). The combined use of T-705 and TD-Borna decreased the BoDV-1 load more efficiently than T-705 or TD-Borna alone (Figure 3B). To confirm this result, we evaluated BoDV-1 infection by western blotting (Figure 3C). Again, both T-705 and TD-Borna efficiently decreased the amount of the N protein and the combined use of T-705 and TD-Borna decreased the protein amount more efficiently than T-705 or TD-Borna alone (Figures 3C,D). These results indicate that the combined use is the most effective way to restrict BoDV-1 infection at this time.

### Reduction in the BoDV-1 Load by TD-Borna in Neuron-Derived Cell Lines

**FIGURE 4:**
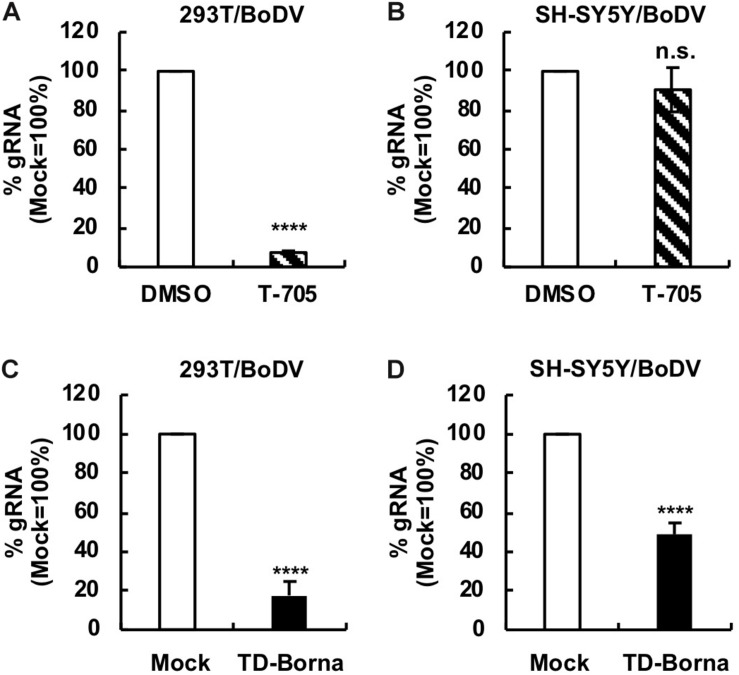
Reduction in the BoDV-1 load by TD-Borna in neuronal and non-neuronal cell lines. **(A,B)** Effect of T-705 on BoDV-1 replication in 293T/BoDV **(A)** and SH-SY5Y/BoDV **(B)** cells. **(C,D)** Effect of TD-Borna on BoDV-1 replication in 293T/BoDV **(C)** and SH-SY5Y/BoDV **(D)** cells. The cells were treated with T-705 **(A,B)** or TD-Borna **(C,D)** for 2 days. The amount of BoDV-1 gRNA in the cells was determined by RT-qPCR analyses. Mock, the scrambled siRNA-treated control. Values are expressed as the mean ± SE of three independent experiments. ^****^P < 0.001 and n.s., no significance (vs. DMSO or mock).

Since BoDV-1 is a highly neurotropic virus, it is important to evaluate the effect of any BoDV-1 therapeutic strategy on BoDV-1 infection in neurons or neuron-derived cells. To this end, we used a human SH-SY5Y neuroblastoma cell line. We established persistently BoDV-1-infected SH-SY5Y (SH-SY5Y/BoDV) cells and treated the cells with T-705, ribavirin, or TD-Borna (Figure 4 and Supplementary Figure S4). T-705 decreased the BoDV-1 load in 293T/BoDV cells (Figure 4A), consistent with the previous study (Tokunaga et al., 2017), but not in SH-SY5Y/BoDV cells (Figure 4B). Ribavirin efficiently decreased the BoDV-1 load in 293T/BoDV cells, whereas it only slightly decreased the load in SH-SY5Y/BoDV (Supplementary Figures S4A,B). These results suggest that T-705 and ribavirin might be less effective for BoDV-1 infection in the central nervous system (CNS). On the other hand, TD-Borna successfully decreased the BoDV-1 load in both 293T/BoDV and SH-SY5Y/BoDV cells (Figures 4C,D). Thus, TD-Borna exhibited the strongest antiviral effect among tested treatments in SH-SY5Y/BoDV cells. To confirm these results, we used another neuron-derived cell line, a mouse N2a neuroblastoma cell line, and established persistently BoDV-1-infected N2a (N2a/BoDV) cells. T-705 did not suppress BoDV-1 in N2a/BoDV cells, ribavirin decreased the load only slightly, and TD-Borna decreased most efficiently (Supplementary Figures S4C, S5), confirming the results in SH-SY5Y/BoDV cells (Figure 4 and Supplementary Figure S4). We further sought to evaluate the effect of the combined use of TD-Borna and ribavirin in SH-SY5Y/BoDV and N2a/BoDV cells. However, the combined use exhibited strong cytotoxicity and we could not evaluate the combined effect on BoDV-1 infection. Taken together, the results demonstrate that TD-Borna may control BoDV infection most efficiently in the CNS.

## Discussion

In this study, we designed several siRNAs targeting BoDV-1 mRNAs, evaluated their efficacy, and developed a novel therapeutic tool, TD-Borna. Because miRNA targeting BoDV-1 antigenomic RNA does not decrease the amount of BoDV-1 antigenomic RNA possibly due to the encapsidation of antigenomic RNA by the N protein (Honda et al., 2016), TD-Borna was expected to target primarily BoDV-1 mRNAs but not BoDV-1 antigenomic RNA. However, we detected a reduction in both BoDV-1 mRNAs and gRNA by TD-Borna treatment (Figures 2D–F). This is reasonable because TD-Borna targeted the N and M/G/L mRNAs, which encode viral proteins indispensable for the synthesis of BoDV-1 gRNA (Tomonaga et al., 2002; Honda and Tomonaga, 2013). Thus, TD-Borna is likely to suppress BoDV-1 by reducing the N and L protein levels.

So far, T-705 and ribavirin have been proposed as antivirals against bornaviruses (Mizutani et al., 1998; Musser et al., 2015; Reuter et al., 2016; Tokunaga et al., 2017). Both antivirals are expected to inhibit viral RNA synthesis by viral RNA-dependent RNA polymerase. T-705 inhibits bornavirus replication more efficiently than ribavirin and has the potential to eliminate bornaviruses from persistently infected cells (Tokunaga et al., 2017). One concern regarding T-705 treatment is that it takes several weeks to control bornavirus infection (Tokunaga et al., 2017). Considering that brain damage is not repairable, this concern should be addressed. TD-Borna is a siRNA cocktail against BoDV-1 that degrades BoDV-1 RNAs. Since TD-Borna suppressed BoDV-1 infection in a different mechanism from that of T-705, we reasoned that a combination therapy of T-705 and TD-Borna would shorten the time to control BoDV-1. As expected, the combination therapy decreased the BoDV-1 load in persistently infected cells most efficiently (Figure 3). We therefore propose a combination therapy with TD-Borna and T-705 as a novel anti-BoDV-1 therapy candidate.

Borna disease virus is a highly neurotropic virus, and acute BoDV-1 infection can cause fatal encephalitis in various animals including humans (Staeheli et al., 2000; Hoffmann et al., 2015; Korn et al., 2018; Schlottau et al., 2018). Although the links between BoDV-1 and CNS disorders are still debated, possible cases of BoDV-1 infection are reported for schizophrenia, major depression, and autism (Nakamura et al., 2000; Wang et al., 2014; Honda et al., 2017b, 2018). Because of these observations, it is pivotal to develop antivirals that exhibit antiviral effects in neuronal cells. We found that T-705 did not suppress BoDV-1 infection in neuron-derived cells, such as SH-SY5Y and N2a cells (Figure 4B and Supplementary Figure S5A), although it was an effective antiviral for BoDV-1 in non-neuronal cells, such as 293T (Figure 4A) and Vero cells (Tokunaga et al., 2017). At present, the reason why T-705 was less effective in neuron-derived cells is not clear and needs to be clarified in future studies. On the other hand, TD-Borna downregulated the BoDV-1 load in all the cell types (Figures 4C,D and Supplementary Figure S5B). These results emphasize the usefulness of TD-Borna in treating BoDV infection in the CNS.

In conclusion, we successfully developed a novel anti-BoDV-1 agent, TD-Borna. Combination therapy of TD-Borna with T-705 exhibited the strongest antiviral effect. Furthermore, TD-Borna can be used to treat BoDV-1 infection in neuronal cells, in which T-705 is less effective. Taken together, TD-Borna will be a useful option to treat BoDV-1 infection in the CNS. Although it is necessary to develop an efficient siRNA delivery system to the CNS and to validate the antiviral effect in an animal model before the practical use of TD-Borna, our study may pave the way for improving the outcome of BoDV-1 infection. In addition, our study also potentially contributes to increasing the safety of BoDV-1-based episomal vectors (REVecs) (Daito et al., 2011; Honda et al., 2016; Yamamoto et al., 2019) in the future by offering a tool to control the REVec load.

## Data Availability Statement

All datasets generated for this study are available on request to the corresponding author.

## Author Contributions

DT, SO, KU, and TH conducted the experiments. DT and TH analyzed the data. TH conceived and designed the study, and wrote the manuscript.

## Conflict of Interest

The authors declare that the research was conducted in the absence of any commercial or financial relationships that could be construed as a potential conflict of interest.
