# Supplementary material for: A Small Interfering RNA Cocktail Targeting the Nucleoprotein and Large Protein Genes Suppresses Borna Disease Virus Infection
Source: Front Microbiol. 2019 Nov 29;10:2781. doi: 10.3389/fmicb.2019.02781 (PMC6895540; doi:10.3389/fmicb.2019.02781)
Supplement: Supplementary file 1 [file Table_1.DOCX]

Table S1. List of primers and probes used in this study.

| Primer | Sequence 5' to 3' | Description |
| --- | --- | --- |
| BoDV-1 gRNA probe | FAM-AGA ACC CCT CCA TGA TCT CAG ACC CAG A-TAMRA | Real-time RT-PCR (Hayashi et al., 2009) |
| BoDV-1 gRNA-specific RT primer for huP2Br strain | TGT TGC GCT AAC AAC AAA CC AAT CAC | Real-time RT-PCR (Hayashi et al., 2009) |
| BoDV-1 gRNA-forward primer for huP2Br strain | ATG CAT TGA CCC AAC CAG TC | Real-time RT-PCR |
| BoDV-1 gRNA-reverse primer for huP2Br strain | ATC ATT CGA CAG CTG CTC CCT TC | Real-time RT-PCR |
| BoDV-1 gRNA-specific RT primer for HOT6 strain (bkn207) | GTT GCG TTA ACA ACA AAC CAA TCA T | Real-time RT-PCR (Honda et al., 2017a) |
| BoDV-1 gRNA-forward primer for HOT6 strain (bkn208) | ATG CAT TGA CCC AAC CGG TA | Real-time RT-PCR (Honda et al., 2017a) |
| BoDV-1 gRNA-reverse primer for HOT6 strain (bkn209) | ATC ATT CGA TAG CTG CTC CCT TC | Real-time RT-PCR (Honda et al., 2017a) |
| BoDV-1 N-forward primer (OU277) | GGA GCC GAG CAG ATC AAG AA | Real-time RT-PCR |
| BoDV-1 N-reverse primer (OU278) | CAC AAA GGA GCC TAC CCA GG | Real-time RT-PCR |
| BoDV-1 L probe | FAM-CGA GGC ATC CGT GGT CAG CAG AT-TAMRA | Real-time RT-PCR |
| BoDV-1 L-forward primer | GGA AGC GCC CCG TGT T | Real-time RT-PCR |
| BoDV-1 L-reverse primer (OU283) | CCC CCA CAG TGA TTC GCT TA | Real-time RT-PCR |
| Human GAPDH-forward primer | CCT GCA CCA CCA ACT GCT TA | Real-time RT-PCR |
| Human GAPDH-reverse primer | GGC CAT CCA CAG TCT TCT GAG | Real-time RT-PCR |
| Mouse GAPDH-forward primer (bkn343) | ACG GCA CAG TCA AGG CCG AG | Real-time RT-PCR |
| Mouse GAPDH-reverse primer (bkn344) | ATC GGC AGA AGG GGC GGA GA | Real-time RT-PCR |
| BoDV-1 N-target forward primer | GTG GAA TTC CGC AAG TTA TGC GCA GAT GA | Sequencing of the target sites |
| BoDV-1 N-target reverse primer | CAC GTC GAC TCG GAG CTC ATC ACA GGT TG | Sequencing of the target sites |
| BoDV-1 L-target forward primer | GTG GAA TTC TAA TCG CCT CAG TGG TCC AG | Sequencing of the target sites |
| BoDV-1 L-target reverse primer | CAC AGA TCT GCT GCC ATC GTC GGT CAA AT | Sequencing of the target sites |
