# Supplementary material for: A Small Interfering RNA Cocktail Targeting the Nucleoprotein and Large Protein Genes Suppresses Borna Disease Virus Infection
Source: Front Microbiol. 2019 Nov 29;10:2781. doi: 10.3389/fmicb.2019.02781 (PMC6895540; doi:10.3389/fmicb.2019.02781)
Supplement: Supplementary file 2 [file Data_Sheet_1.pdf]

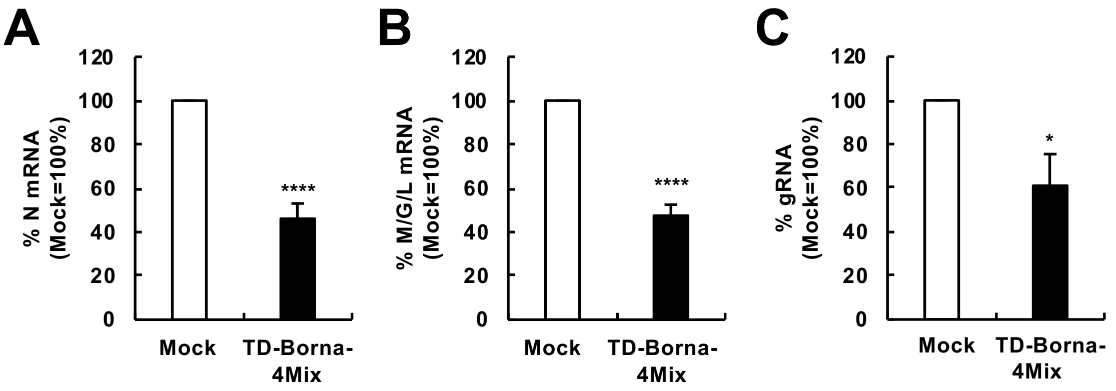

Supplementary Figure S1. Reduction in the BoDV-1 load by the siRNA cocktail, TD-Borna-4Mix, targeting N and M/G/L mRNAs.

(A, B) Effects of the siRNA cocktail, TD-Borna-4Mix, targeting BoDV-1 N and M/G/L mRNAs on BoDV-1 mRNAs. 293T/BoDV cells were treated with TD-Borna-4Mix for 2 days. The amounts of N (A) and M/G/L (B) mRNAs in 293T/BoDV cells were determined by RT-qPCR analyses. (C) Effects of TD-Borna-4Mix on BoDV-1 replication. The amount of BoDV-1 gRNA in 293T/BoDV cells was determined by RT-qPCR analyses. Mock, the scrambled siRNA-treated control. Values are expressed as the mean + S.E. of three independent experiments. *, $P < 0.05$; ****, $P < 0.001$ (vs. mock).

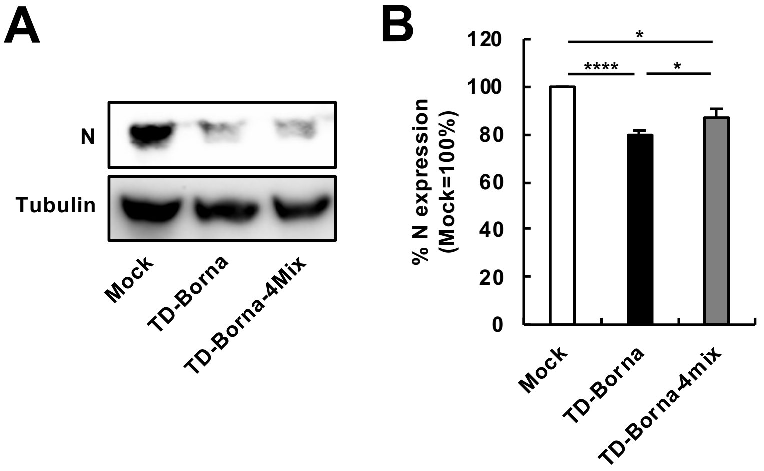

Supplementary Figure S2. Reduction in the amount of the N protein by TD-Borna.
(A) The amount of the N protein in 293T/BoDV cells treated with TD-Borna or TD-Borna-4Mix for 2 days was determined by western blotting using anti-N and anti-tubulin antibodies. (B) Quantification of the amount of the N protein in (A). The band intensity of the N protein in each sample was normalized with that of tubulin. Mock, the scrambled siRNA-treated control. Values are expressed as the mean + S.E. of three independent experiments. *, $P < 0.05$; ****, $P < 0.001$ (vs. mock).

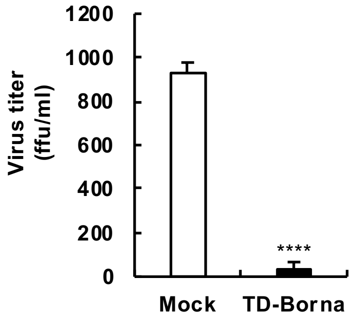

Supplementary Figure S3. Reduction in the production of infectious BoDV-1 particles by TD-Borna. 293T/BoDV cells were treated with TD-Borna for 2 days. The viral titer (focus forming unit/ml, ffu/ml) of viral stocks derived from mock- or TD-Borna-treated 293T/BoDV cells was determined by IFA. Mock, the scrambled siRNA-treated control. Values are expressed as the mean + S.E. of three independent experiments. ****, $P < 0.001$ (vs. mock).

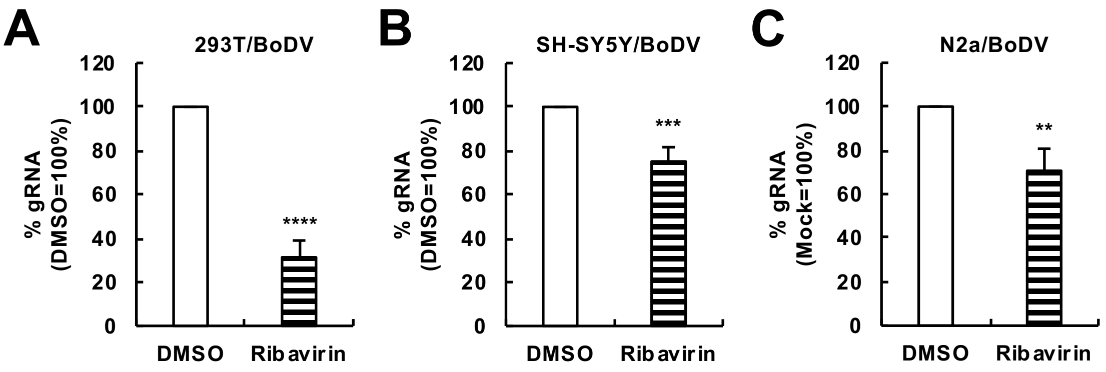

Supplementary Figure S4. Reduction in the BoDV-1 load by ribavirin.
Effects of ribavirin on BoDV-1 replication in 293T/BoDV (A), SH-SY5Y/BoDV (B), and N2a/BoDV cells (C). The amount of BoDV-1 gRNA in the cells was determined by RT-qPCR analyses. Values are expressed as the mean + S.E. of three independent experiments. **, $P < 0.01$; ***, $P < 0.005$; ****, $P < 0.001$ (vs. DMSO).

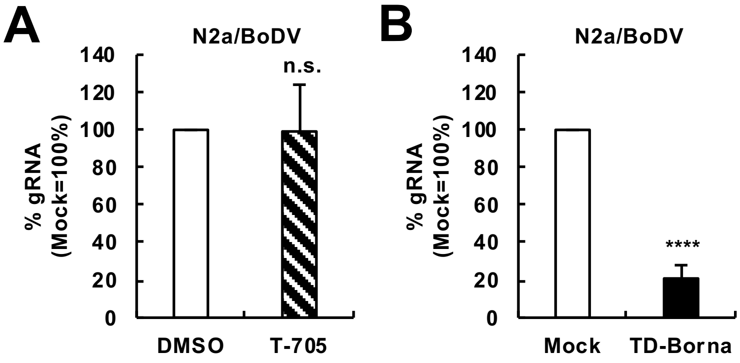

Supplementary Figure S5. Reduction in the BoDV-1 load by TD-Borna in N2a/BoDV cells.
(A, B) Effect of T-705 (A) or TD-Borna (B) on BoDV-1 replication in N2a/BoDV cells. N2a/BoDV cells were treated with T-705 (A) or TD-Borna (B) for 2 days. The amount of BoDV-1 gRNA in N2a/BoDV cells was determined by RT-qPCR analyses. Mock, the scrambled siRNA-treated control. Values are expressed as the mean + S.E. of three independent experiments. ****, $P < 0.001$; n.s., no significance (vs. DMSO or mock).
